# Spiro­[cyclo­propane-1,3′-indolin]-2′-one

**DOI:** 10.1107/S1600536811034167

**Published:** 2011-08-27

**Authors:** Maosen Yuan, Qi Wang, Yuejun Zhang, Junru Wang

**Affiliations:** a College of Science, Northwest A&F University, Yangling 712100, Shannxi Province, People’s Republic of China

## Abstract

In the title mol­ecule, C_10_H_9_NO, the dihedral angle between the mean plane of the cyclo­propane ring and the essentially planar [maximum deviation = 0.032 (2) Å] indole ring system is 87.65 (17)°. In the crystal, inter­molecular N—H⋯O hydrogen bonds link mol­ecules into one-dimensional chains along [100].

## Related literature

For the applications of indoline-2-one and its derivatives, see: Wang *et al.* (2011 ▶); Ji *et al.* (2010 ▶). For a related structure, see: Yong *et al.* (2007 ▶).
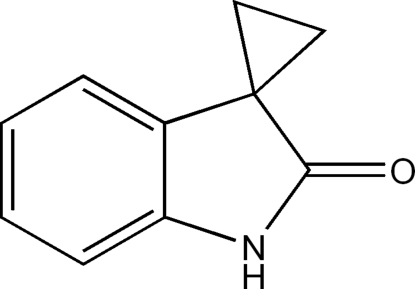


## Experimental

### 

#### Crystal data

C_10_H_9_NO
*M* *_r_* = 159.18
Orthorhombic, 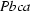

*a* = 7.4348 (6) Å
*b* = 14.0589 (11) Å
*c* = 15.6401 (16) Å
*V* = 1634.8 (2) Å^3^
*Z* = 8
Mo *K*α radiation
μ = 0.09 mm^−1^
*T* = 298 K
0.50 × 0.45 × 0.42 mm

#### Data collection

Bruker SMART CCD diffractometer
Absorption correction: multi-scan (*SADABS*; Sheldrick, 1996 ▶) *T*_min_ = 0.959, *T*_max_ = 0.965
7724 measured reflections
1442 independent reflections
1024 reflections with *I* > 2σ(*I*)
*R*_int_ = 0.033

#### Refinement

*R*[*F* ^2^ > 2σ(*F* ^2^)] = 0.035
*wR*(*F* ^2^) = 0.106
*S* = 1.09
1442 reflections
110 parameters
H-atom parameters constrained
Δρ_max_ = 0.15 e Å^−3^
Δρ_min_ = −0.11 e Å^−3^

### 

Data collection: *SMART* (Bruker, 2001 ▶); cell refinement: *SAINT* (Bruker, 2001 ▶); data reduction: *SAINT*; program(s) used to solve structure: *SHELXS97* (Sheldrick, 2008 ▶); program(s) used to refine structure: *SHELXL97* (Sheldrick, 2008 ▶); molecular graphics: *SHELXTL* (Sheldrick, 2008 ▶) and *PLATON* (Spek, 2009 ▶); software used to prepare material for publication: *SHELXTL*.

## Supplementary Material

Crystal structure: contains datablock(s) global, I. DOI: 10.1107/S1600536811034167/lh5317sup1.cif

Structure factors: contains datablock(s) I. DOI: 10.1107/S1600536811034167/lh5317Isup2.hkl

Supplementary material file. DOI: 10.1107/S1600536811034167/lh5317Isup3.cml

Additional supplementary materials:  crystallographic information; 3D view; checkCIF report

## Figures and Tables

**Table 1:** Hydrogen-bond geometry (Å, °)

*D*—H⋯*A*	*D*—H	H⋯*A*	*D*⋯*A*	*D*—H⋯*A*
N1—H1⋯O1^i^	0.86	2.00	2.855 (2)	170

Symmetry code: (i) 
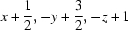
.

**Fig. 1.**
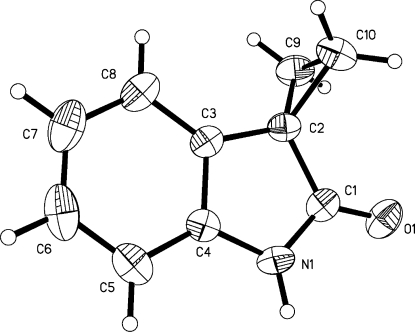
The molecular structure of the title compound with the atom numbering scheme. Displacement ellipsoids are drawn at the 30% probability level.

**Fig. 2.**
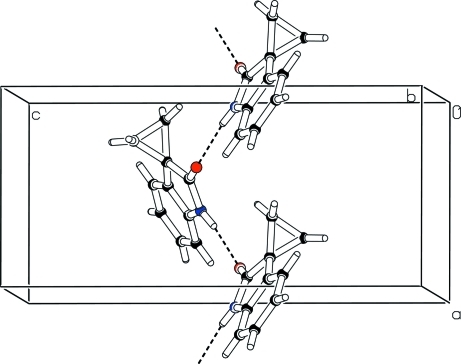
Part of the crystal structure with hydrogen bonds shown as dashed lines.

## supplementary crystallographic information

### Comment

Indoline-2-one and its derivatives have been widely explored as materials for the synthesis of antitumor agents (Wang *et al.*, 2011). Indoline-2-one may also be used as a precursor for synthesizing organic luminescent molecules because of its perfect conformation (Ji *et al.*, 2010). In the course of exploring new electro-optic compounds, we obtained the title compound compound and the crystal structure is reported herein.

The title compound is a spiro-compound and has two substituent ring systems, an indoline-2-one ring and a cyclopropane ring which share the sprio atom C2 (Fig. 1). The dihedral angle between the two rings is 87.65 (17)°. The crystal structure of a similar compound ethyl(1'R,2'R)-2-oxo-1,2-dihydrospiro (cyclopropane-1',3-indole)-2'-carboxylate has been published (Yong *et al.* 2007). In the crystal, intermolecular N—H···O hydrogen bonds link molecules into one-dimensional chains along [100] (Fig. 2).

### Experimental

Indolin-2-one (0.50 g, 3.76 mmol) was dissolved in THF (20 mL) and KOH (0.80 g, 14.3 mmol) was slowly added. After heating the stirred mixture at reflux temperature for 30 min, a solution of 1,2-dibromoethane (1.00 g, 5.35 mmol) in THF was slowly added and the refluxing continued for 2 h. The mixture was then cooled to 333 K and poured into water (200 mL) and was extracted with chloroform and dried over Na_2_SO_4_. After removing the solvent, the crude product was purified by column chromatography on silica gel, affording the title compound (yield: 0.18 g, 30%). The compound was then dissolved in THF, and colorless crystals were formed on slow evaporation at room temperature over one week.

### Refinement

All H atoms were placed in geometrically calculated positions and refined using a riding model with C—H = 0.93-0.97 Å and N—H = 0.86 Å and with *U*_iso_(H) = 1.2*U*_eq_(C, N).

### Crystal data

**Table:**

C_10_H_9_NO	*F*(000) = 672
*M**_r_* = 159.18	*D*_x_ = 1.294 Mg m^−^^3^
Orthorhombic, *P**b**c**a*	Mo *K*α radiation, λ = 0.71073 Å
Hall symbol: -P 2ac 2ab	Cell parameters from 2492 reflections
*a* = 7.4348 (6) Å	θ = 2.9–26.3°
*b* = 14.0589 (11) Å	µ = 0.09 mm^−^^1^
*c* = 15.6401 (16) Å	*T* = 298 K
*V* = 1634.8 (2) Å^3^	Block, colorless
*Z* = 8	0.50 × 0.45 × 0.42 mm

### Data collection

**Table:**

Bruker SMART CCD diffractometer	1442 independent reflections
Radiation source: fine-focus sealed tube	1024 reflections with *I* > 2σ(*I*)
graphite	*R*_int_ = 0.033
φ and ω scans	θ_max_ = 25.0°, θ_min_ = 2.9°
Absorption correction: multi-scan (*SADABS*; Sheldrick, 1996)	*h* = −8→5
*T*_min_ = 0.959, *T*_max_ = 0.965	*k* = −16→16
7724 measured reflections	*l* = −18→18

### Refinement

**Table:**

Refinement on *F*^2^	Secondary atom site location: difference Fourier map
Least-squares matrix: full	Hydrogen site location: inferred from neighbouring sites
*R*[*F*^2^ > 2σ(*F*^2^)] = 0.035	H-atom parameters constrained
*wR*(*F*^2^) = 0.106	*w* = 1/[σ^2^(*F*_o_^2^) + (0.039*P*)^2^ + 0.514*P*] where *P* = (*F*_o_^2^ + 2*F*_c_^2^)/3
*S* = 1.09	(Δ/σ)_max_ < 0.001
1442 reflections	Δρ_max_ = 0.15 e Å^−^^3^
110 parameters	Δρ_min_ = −0.11 e Å^−^^3^
0 restraints	Extinction correction: *SHELXL97*, Fc^*^=kFc[1+0.001xFc^2^λ^3^/sin(2θ)]^-1/4^
Primary atom site location: structure-invariant direct methods	Extinction coefficient: 0.029 (3)

### Special details

**Table:**

Geometry. All esds (except the esd in the dihedral angle between two l.s. planes) are estimated using the full covariance matrix. The cell esds are taken into account individually in the estimation of esds in distances, angles and torsion angles; correlations between esds in cell parameters are only used when they are defined by crystal symmetry. An approximate (isotropic) treatment of cell esds is used for estimating esds involving l.s. planes.
Refinement. Refinement of F^2^ against ALL reflections. The weighted R-factor wR and goodness of fit S are based on F^2^, conventional R-factors R are based on F, with F set to zero for negative F^2^. The threshold expression of F^2^ > 2sigma(F^2^) is used only for calculating R-factors(gt) etc. and is not relevant to the choice of reflections for refinement. R-factors based on F^2^ are statistically about twice as large as those based on F, and R- factors based on ALL data will be even larger.

### Fractional atomic coordinates and isotropic or equivalent isotropic displacement parameters (Å^2^)

**Table:**

	*x*	*y*	*z*	*U*_iso_*/*U*_eq_	
N1	0.59216 (19)	0.65349 (11)	0.54786 (9)	0.0505 (4)	
H1	0.6712	0.6798	0.5154	0.061*	
O1	0.38643 (18)	0.77529 (9)	0.55038 (10)	0.0683 (5)	
C1	0.4374 (2)	0.69564 (13)	0.57235 (12)	0.0485 (5)	
C2	0.3453 (2)	0.62839 (13)	0.63111 (11)	0.0457 (5)	
C3	0.4605 (2)	0.54356 (12)	0.63342 (11)	0.0445 (5)	
C4	0.6083 (2)	0.56211 (12)	0.58160 (11)	0.0440 (4)	
C5	0.7419 (3)	0.49602 (16)	0.56891 (13)	0.0617 (6)	
H5	0.8403	0.5091	0.5341	0.074*	
C6	0.7245 (4)	0.40906 (16)	0.60998 (17)	0.0771 (7)	
H6	0.8131	0.3630	0.6029	0.092*	
C7	0.5787 (4)	0.38995 (16)	0.66098 (17)	0.0797 (8)	
H7	0.5697	0.3311	0.6877	0.096*	
C8	0.4454 (3)	0.45660 (14)	0.67318 (14)	0.0651 (6)	
H8	0.3468	0.4431	0.7077	0.078*	
C9	0.1417 (2)	0.62741 (16)	0.63580 (14)	0.0625 (6)	
H9A	0.0765	0.6704	0.5985	0.075*	
H9B	0.0831	0.5668	0.6456	0.075*	
C10	0.2446 (3)	0.66937 (17)	0.70712 (13)	0.0663 (6)	
H10A	0.2490	0.6343	0.7605	0.080*	
H10B	0.2424	0.7380	0.7134	0.080*	

### Atomic displacement parameters (Å^2^)

**Table:**

	*U*^11^	*U*^22^	*U*^33^	*U*^12^	*U*^13^	*U*^23^
N1	0.0398 (8)	0.0637 (10)	0.0481 (9)	−0.0077 (7)	0.0010 (7)	0.0114 (7)
O1	0.0568 (9)	0.0588 (9)	0.0892 (11)	−0.0017 (7)	−0.0157 (8)	0.0200 (8)
C1	0.0412 (10)	0.0541 (11)	0.0502 (11)	−0.0046 (8)	−0.0123 (8)	0.0059 (9)
C2	0.0391 (10)	0.0552 (11)	0.0429 (10)	−0.0060 (8)	−0.0023 (8)	0.0017 (8)
C3	0.0453 (10)	0.0490 (10)	0.0394 (10)	−0.0077 (8)	−0.0089 (8)	0.0019 (8)
C4	0.0402 (9)	0.0532 (10)	0.0386 (9)	−0.0024 (8)	−0.0092 (8)	−0.0033 (8)
C5	0.0459 (11)	0.0789 (14)	0.0602 (13)	0.0048 (10)	−0.0129 (9)	−0.0202 (11)
C6	0.0754 (16)	0.0609 (14)	0.0949 (18)	0.0185 (12)	−0.0395 (15)	−0.0222 (13)
C7	0.0890 (19)	0.0562 (13)	0.0939 (18)	−0.0028 (13)	−0.0356 (16)	0.0110 (12)
C8	0.0685 (14)	0.0618 (13)	0.0651 (14)	−0.0127 (11)	−0.0119 (11)	0.0146 (11)
C9	0.0403 (11)	0.0805 (14)	0.0668 (14)	−0.0067 (9)	0.0014 (10)	0.0049 (11)
C10	0.0555 (12)	0.0875 (15)	0.0558 (12)	0.0020 (11)	0.0062 (10)	−0.0075 (11)

### Geometric parameters (Å, °)

**Table:**

N1—C1	1.350 (2)	C5—H5	0.9300
N1—C4	1.394 (2)	C6—C7	1.373 (4)
N1—H1	0.8600	C6—H6	0.9300
O1—C1	1.231 (2)	C7—C8	1.377 (3)
C1—C2	1.486 (2)	C7—H7	0.9300
C2—C3	1.469 (2)	C8—H8	0.9300
C2—C9	1.515 (3)	C9—C10	1.476 (3)
C2—C10	1.518 (3)	C9—H9A	0.9700
C3—C8	1.376 (2)	C9—H9B	0.9700
C3—C4	1.390 (2)	C10—H10A	0.9700
C4—C5	1.375 (3)	C10—H10B	0.9700
C5—C6	1.387 (3)		
			
C1—N1—C4	111.75 (15)	C7—C6—C5	121.0 (2)
C1—N1—H1	124.1	C7—C6—H6	119.5
C4—N1—H1	124.1	C5—C6—H6	119.5
O1—C1—N1	125.62 (18)	C6—C7—C8	121.1 (2)
O1—C1—C2	127.56 (18)	C6—C7—H7	119.5
N1—C1—C2	106.81 (15)	C8—C7—H7	119.5
C3—C2—C1	105.25 (15)	C3—C8—C7	118.9 (2)
C3—C2—C9	125.03 (17)	C3—C8—H8	120.6
C1—C2—C9	119.74 (16)	C7—C8—H8	120.6
C3—C2—C10	125.19 (17)	C10—C9—C2	60.99 (13)
C1—C2—C10	118.04 (16)	C10—C9—H9A	117.7
C9—C2—C10	58.22 (13)	C2—C9—H9A	117.7
C8—C3—C4	119.65 (18)	C10—C9—H9B	117.7
C8—C3—C2	133.21 (18)	C2—C9—H9B	117.7
C4—C3—C2	107.12 (15)	H9A—C9—H9B	114.8
C5—C4—C3	121.91 (18)	C9—C10—C2	60.79 (13)
C5—C4—N1	129.09 (18)	C9—C10—H10A	117.7
C3—C4—N1	109.00 (15)	C2—C10—H10A	117.7
C4—C5—C6	117.5 (2)	C9—C10—H10B	117.7
C4—C5—H5	121.3	C2—C10—H10B	117.7
C6—C5—H5	121.3	H10A—C10—H10B	114.8
			
C4—N1—C1—O1	−178.13 (17)	C8—C3—C4—N1	179.15 (16)
C4—N1—C1—C2	2.87 (19)	C2—C3—C4—N1	0.42 (18)
O1—C1—C2—C3	178.55 (18)	C1—N1—C4—C5	177.34 (17)
N1—C1—C2—C3	−2.47 (18)	C1—N1—C4—C3	−2.14 (19)
O1—C1—C2—C9	31.3 (3)	C3—C4—C5—C6	0.0 (3)
N1—C1—C2—C9	−149.71 (17)	N1—C4—C5—C6	−179.43 (17)
O1—C1—C2—C10	−36.2 (3)	C4—C5—C6—C7	0.3 (3)
N1—C1—C2—C10	142.81 (16)	C5—C6—C7—C8	−0.2 (3)
C1—C2—C3—C8	−177.26 (19)	C4—C3—C8—C7	0.5 (3)
C9—C2—C3—C8	−32.3 (3)	C2—C3—C8—C7	178.80 (19)
C10—C2—C3—C8	40.7 (3)	C6—C7—C8—C3	−0.2 (3)
C1—C2—C3—C4	1.23 (18)	C3—C2—C9—C10	113.2 (2)
C9—C2—C3—C4	146.22 (18)	C1—C2—C9—C10	−106.4 (2)
C10—C2—C3—C4	−140.81 (17)	C3—C2—C10—C9	−112.9 (2)
C8—C3—C4—C5	−0.4 (3)	C1—C2—C10—C9	109.34 (19)
C2—C3—C4—C5	−179.11 (16)		

### Hydrogen-bond geometry (Å, °)

**Table:**

*D*—H···*A*	*D*—H	H···*A*	*D*···*A*	*D*—H···*A*
N1—H1···O1^i^	0.86	2.00	2.855 (2)	170

Symmetry codes: (i) *x*+1/2, −*y*+3/2, −*z*+1.
